# PB.29. Retrospective correlation between MRI and histopathology in preoperative assessment of invasive breast carcinoma

**DOI:** 10.1186/bcr3726

**Published:** 2014-11-03

**Authors:** G Gracey, J McKillen, C Majury, J McAllister

**Affiliations:** 1 Ulster Hospital, Dundonald, UK

## Introduction

Accurate preoperative assessment of a breast tumor has an important role in the planning of breast cancer treatment. The purpose of this study was to assess the accuracy of preoperative MRI in sizing breast tumors of varying pathological subtypes.

## Methods

A patient list was populated from the local PACS database. The sample included all patients with a new diagnosis of breast cancer who underwent preoperative contrast-enhanced MRI breast imaging over an 18-month period. The MRI breast images were reviewed and the largest dimension (mm) was recorded. Correlation with the postoperative pathology report was made.

## Results

The sample size included 42 patients with 43 breast cancers. Average tumor size was 42 mm. There was direct MRI/pathological size correlation in 41% of cases. MRI overestimated 19% of tumor sizes, and underestimated 40%. DCIS was under estimated in 40%. MRI accurately sized 62% of intermediate DCIS compared with 25% high-grade DCIS. LCIS was underestimated in 31%. MRI accurately sized 45% of invasive ductal carcinoma and 40% of invasive lobular carcinoma.

## Conclusion

This study shows that MRI accurately sizes breast cancer tumors in 41% of cases, overestimates 19% and underestimates 40%. MRI is more accurate at measuring intermediate DCIS compared with high-grade DCIS. A similar accuracy is found between IDC and ILC. Better preoperative tumor sizing is fundamental for initial surgical planning, decreasing the need for re-excision or mastectomy and therefore appropriate treatment.

